# P-892. Impact of early continuous renal replacement therapy in patients with septic acute kidney injury: an analysis of the MIMIC-IV database

**DOI:** 10.1093/ofid/ofae631.1083

**Published:** 2025-01-29

**Authors:** Yongseop Lee, Jun Hye Seo, Jaeeun Seong, Sangmin Ahn, Min Han, Jung Ah Lee, Jung Ho Kim, Jin Young Ahn, Su Jin Jeong, Jun Yong Choi, Joon-sup Yeom, Hyung Jung Oh, Nam Su Ku

**Affiliations:** Division of Infectious Diseases, Department of Internal Medicine and AIDS Research Institute, Yonsei University College of Medicine, Seodaemun-gu, Seoul-t'ukpyolsi, Republic of Korea; 2Division of Nephrology, Department of Internal Medicine, National Health Insurance Service Ilsan Hospital, Goyang-si, Kangwon-do, Republic of Korea; Division of Infectious Diseases, Department of Internal Medicine and AIDS Research Institute, Yonsei University College of Medicine, Seodaemun-gu, Seoul-t'ukpyolsi, Republic of Korea; Yonsei University College of Medicine, seoul, Seoul-t'ukpyolsi, Republic of Korea; Yonsei University School of Medicine, Seoul, Seoul-t'ukpyolsi, Republic of Korea; Yonsei University College of Medicine, seoul, Seoul-t'ukpyolsi, Republic of Korea; Yonsei University College of Medicine, seoul, Seoul-t'ukpyolsi, Republic of Korea; Yonsei University College of Medicine, seoul, Seoul-t'ukpyolsi, Republic of Korea; Yonsei University College of Medicine, seoul, Seoul-t'ukpyolsi, Republic of Korea; Yonsei University College of Medicine, seoul, Seoul-t'ukpyolsi, Republic of Korea; Division of Infectious Diseases, Department of Internal Medicine, Yonsei University College of Medicine, Seoul, Seoul-t'ukpyolsi, Republic of Korea; 3Division of Nephrology, Sheikh Khalifa Specialty Hospital, Al Shohadaa Road, Exit 119, Ras Al Khaimah, United Arab Emirates; Division of Infectious Diseases, Department of Internal Medicine, Yonsei University College of Medicine, Seoul, Seoul-t'ukpyolsi, Republic of Korea

## Abstract

**Background:**

Renal replacement therapy (RRT) is an important treatment option for septic acute kidney injury (AKI); however, the optimal timing for its initiation remains controversial. Here, we aimed to investigate the clinical outcomes of early continuous renal replacement therapy (CRRT), which was defined as CRRT initiation within 6 hours of the onset of septic AKI, which is earlier than the time of initiation defined in previous studies.

**Figure 1.**
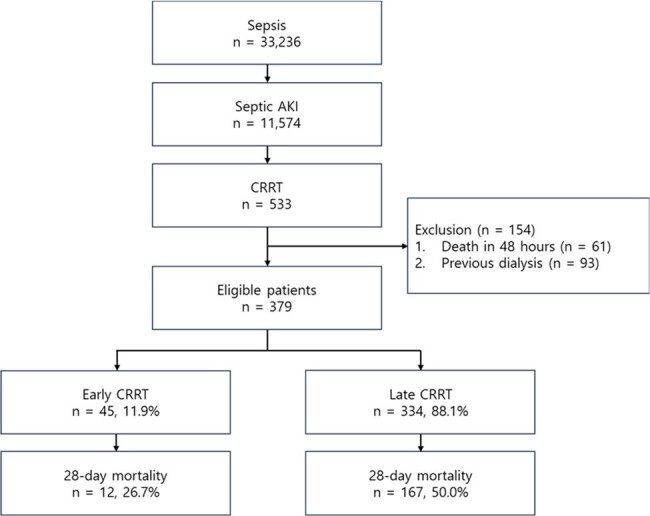
Study flowchart.

**Methods:**

We used clinical data sourced from the Medical Information Mart for Intensive Care IV (MIMIC-IV) database. This study included patients aged ≥18 years who met the sepsis diagnostic criteria and received CRRT because of stage 2 or 3 AKI. Early and late CRRT were defined as CRRT initiation within 6 hours and after 6 hours of the development of septic AKI, respectively.

**Table 1.**
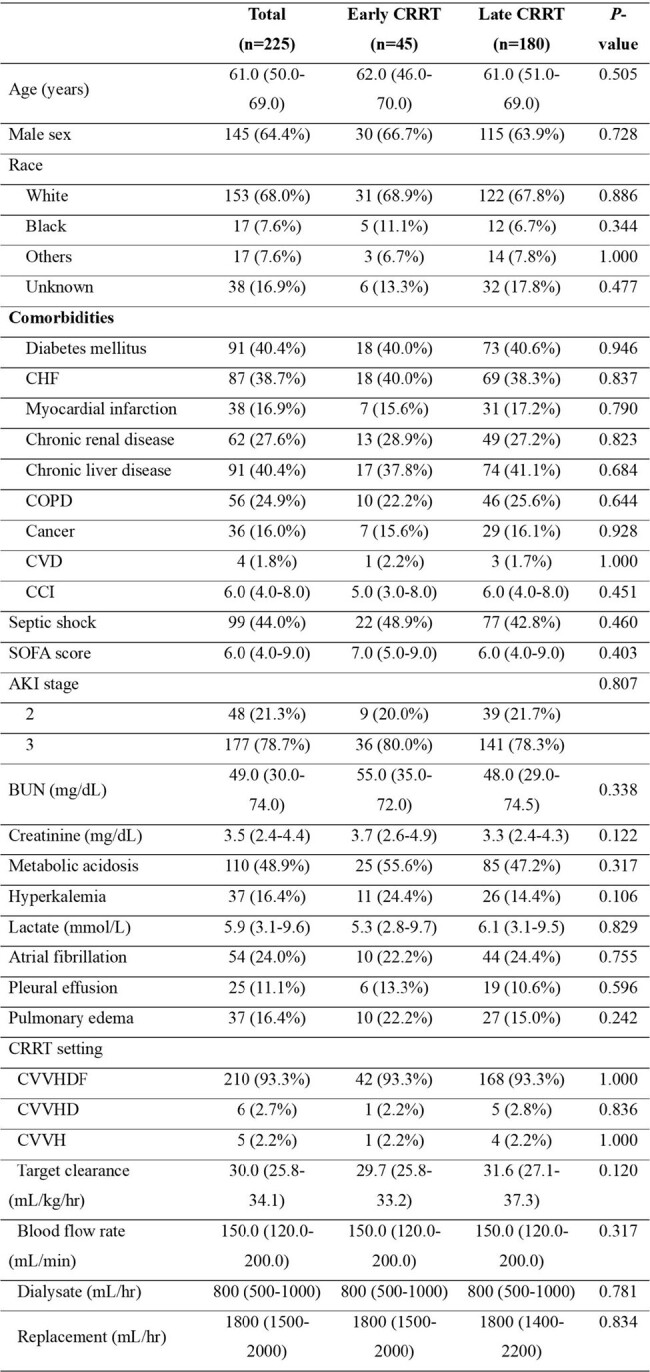
Clinical characteristics of patients with sepsis after propensity matching

**Results:**

Of the 33,236 patients diagnosed with sepsis, 553 underwent CRRT for septic AKI. After excluding cases of early mortality and patients with a history of dialysis, 45 and 334 patients were included in the early and late CRRT groups, respectively. After propensity score matching, the 28-day mortality rate was significantly lower in the early CRRT group than that in the late CRRT group (26.7% vs. 43.9%, *P*=0.035). The early CRRT group also had a significantly greater number of days free of mechanical ventilation (median days, 19; interquartile range [IQR] 3–25) and vasopressor administration (median days, 21, IQR 5–26) than did the late CRRT group (median days, 10.5; IQR, 0–23; *P*=0.037 and median days, 13.5; IQR, 0–25; *P*=0.028, respectively). The Kaplan–Meier curve also showed that early CRRT initiation was associated with an improved 28-day mortality rate (log-rank test, *P*=0.040). In contrast, there was no significant difference in the 28-day mortality between patients who started CRRT within 12 h and those who did not (log-rank test, *P*=0.237).

**Table 2.**
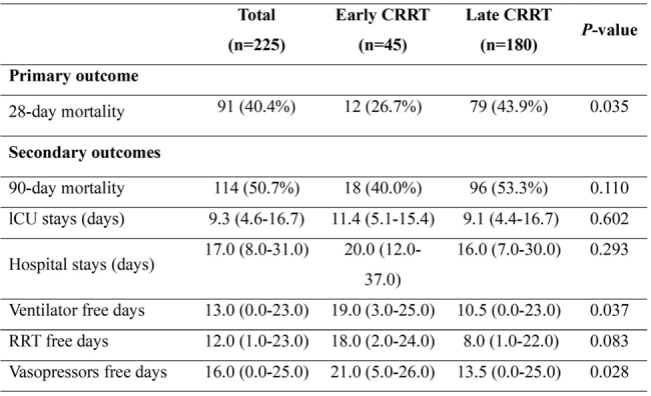
Clinical outcomes of patients with sepsis after propensity score matching

**Conclusion:**

Early CRRT initiation can improve the survival of patients with septic AKI. CRRT must be initiated as early as possible after the onset of septic AKI, preferably within 6 hours.

**Figure 2.**
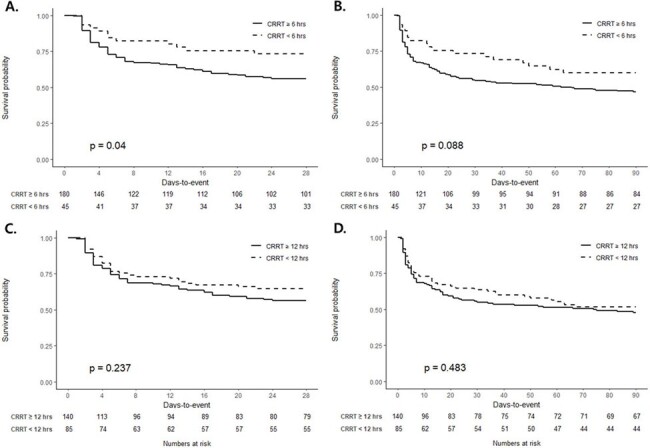
Kaplan-Meier survival curves of patients with septic acute kidney injury after propensity score matching. A. Comparison of 28-day survival and B. 90-day mortality among patients with sepsis between continuous renal replacement therapy initiation < 6 hours and ≥ 6 hours. C. Comparison of 28-day survival and D. 90-day mortality among patients with sepsis between continuous renal replacement therapy initiation < 12 hours and ≥ 12 hours.

**Disclosures:**

**All Authors**: No reported disclosures

